# Bioinformatic analysis and preliminary validation of potential therapeutic targets for COVID-19 infection in asthma patients

**DOI:** 10.1186/s12964-022-01010-2

**Published:** 2022-12-27

**Authors:** Yue Li, Ye Liu, Mengjie Duo, Ruhao Wu, Tianci Jiang, Pengfei Li, Yu Wang, Zhe Cheng

**Affiliations:** Department of Pulmonary and Critical Care Medicine, The First Affiliated Hospital of Zhengzhou University, Zhengzhou, 450052 Henan China

**Keywords:** COVID-19, Asthma, Bioinformatics, Diagnosis, Biomarker

## Abstract

**Background:**

Severe acute respiratory syndrome coronavirus 2 causes coronavirus disease 19 (COVID-19). The number of confirmed cases of COVID-19 is also rapidly increasing worldwide, posing a significant challenge to human safety. Asthma is a risk factor for COVID-19, but the underlying molecular mechanisms of the asthma–COVID-19 interaction remain unclear.

**Methods:**

We used transcriptome analysis to discover molecular biomarkers common to asthma and COVID-19. Gene Expression Omnibus database RNA-seq datasets (GSE195599 and GSE196822) were used to identify differentially expressed genes (DEGs) in asthma and COVID-19 patients. After intersecting the differentially expressed mRNAs, Gene Ontology (GO) and Kyoto Encyclopedia of Genes and Genomes (KEGG) enrichment analyses were performed to identify the common pathogenic molecular mechanism. Bioinformatic methods were used to construct protein–protein interaction (PPI) networks and identify key genes from the networks. An online database was used to predict interactions between transcription factors and key genes. The differentially expressed long noncoding RNAs (lncRNAs) in the GSE195599 and GSE196822 datasets were intersected to construct a competing endogenous RNA (ceRNA) regulatory network. Interaction networks were constructed for key genes with RNA-binding proteins (RBPs) and oxidative stress-related proteins. The diagnostic efficacy of key genes in COVID-19 was verified with the GSE171110 dataset. The differential expression of key genes in asthma was verified with the GSE69683 dataset. An asthma cell model was established with interleukins (IL-4, IL-13 and IL-17A) and transfected with siRNA-CXCR1. The role of CXCR1 in asthma development was preliminarily confirmed.

**Results:**

By intersecting the differentially expressed genes for COVID-19 and asthma, 393 common DEGs were obtained. GO and KEGG enrichment analyses of the DEGs showed that they mainly affected inflammation-, cytokine- and immune-related functions and inflammation-related signaling pathways. By analyzing the PPI network, we obtained 10 key genes: TLR4, TLR2, MMP9, EGF, HCK, FCGR2A, SELP, NFKBIA, CXCR1, and SELL. By intersecting the differentially expressed lncRNAs for COVID-19 and asthma, 13 common differentially expressed lncRNAs were obtained. LncRNAs that regulated microRNAs (miRNAs) were mainly concentrated in intercellular signal transduction, apoptosis, immunity and other related functional pathways. The ceRNA network suggested that there were a variety of regulatory miRNAs and lncRNAs upstream of the key genes. The key genes could also bind a variety of RBPs and oxidative stress-related genes. The key genes also had good diagnostic value in the verification set. In the validation set, the expression of key genes was statistically significant in both the COVID-19 group and the asthma group compared with the healthy control group. CXCR1 expression was upregulated in asthma cell models, and interference with CXCR1 expression significantly reduced cell viability.

**Conclusions:**

Key genes may become diagnostic and predictive biomarkers of outcomes in COVID-19 and asthma.

Video Abstract

**Supplementary Information:**

The online version contains supplementary material available at 10.1186/s12964-022-01010-2.

## Introduction

Coronavirus disease 19 (COVID-19) is the disease caused by novel coronavirus infection [1]. Common symptoms of infection include fever, fatigue, dry cough and shortness of breath [2]. Severe cases can involve acute respiratory distress syndrome, septic shock, refractory metabolic acidosis, and coagulation disorders [3]. Asthma is a common chronic inflammatory disease of the airways. Its main pathological features are airway inflammation and remodeling, which are mainly caused by the long-term accumulation and interactions of inflammatory cells and airway inflammatory factors [4]. Asthma patients are highly susceptible to rhinovirus and influenza virus, which are associated with insufficient interferon production and an insufficient innate immune response to the virus [5]. The novel coronavirus is now a new virus, so people with asthma should take more precautions than healthy people. There have been investigations into the relationship between COVID-19 and asthma. Large population-based cohort studies suggested that adults with asthma were at an increased risk of severe COVID-19, driven by an increased risk of nonallergic asthma. In contrast, patients with allergic asthma did not have a significantly increased risk of severe COVID-19. The study also showed no association between existing asthma genetic polygenic scores and COVID-19 [6]. A study showed that patients with chronic airway disease, once complicated with novel coronavirus pneumonia, had a much higher rate of severe disease and fatality than other diagnosed patients [7]. However, there are also data that do not support the conclusion that asthma patients infected with the novel coronavirus are prone to severe pneumonia [8]. The differences in the existing studies suggest that the interaction between COVID-19 and asthma remains unclear and requires high-quality evidence-based medical study on a large scale [9]. There is still little research on whether COVID-19 and asthma share pathogenic mechanisms and whether the diagnostic and therapeutic biomarkers are the same [10].

The incubation period for COVID-19 is usually 4–8 days, with a maximum range of 1–14 days [11]. In people who already have an underlying disease, SARS-CoV-2 attacks the lungs and many organs of the body due to weakened immunity. The traditional diagnosis of asthma is based primarily on clinical symptoms, lung function tests, or peak flow measurements [12]. Some clinical manifestations of asthma are nonspecific, and lung function tests in some patients show normal results or nonsignificant changes. Biomarkers provide a promising noninvasive method for asthma diagnosis and performance assessment [13]. However, due to issues related to sensitivity and specificity, there is currently no approved clinical marker, and no biomarkers have been included in the asthma treatment guidelines [14]. With the development of biomedicine, asthma research is developing from a focus on clinical symptoms, clinical phenotypes, lung function and drug response to efforts related to genomics, proteomics, and epigenetics [15]. Through a chip and mass spectrometry technology platform, which allows study of the signaling pathways and interacting molecules related to asthma pathophysiology with systematic data integration and analysis, more key molecules will be discovered, and more biomarkers will be identified [16]. Ultimately, personalized and precise treatment of asthma can be realized, providing patients with more effective and safer treatment methods.

Long noncoding RNAs (lncRNAs) are noncoding RNAs over 200 nucleotides in length that regulate gene function at the epigenetic, transcriptional, or posttranscriptional processing level [17]. Studies have shown that lncRNAs play important roles in cell activity and participate in multigene regulatory networks, suggesting that lncRNAs may be promising biomarkers for the early screening and diagnosis of diseases [18]. MicroRNAs (miRNAs) are small noncoding RNA transcripts consisting of 20 nucleotides [19]. The theory of competing endogenous RNA (ceRNA) suggests that endogenous RNAs share the same binding sites as specific miRNAs. These endogenous RNAs can competitively bind to shared miRNAs, impair miRNA repression of target genes, and thus participate in disease progression [20].

In this study, common differentially expressed genes in asthma and COVID-19 were obtained by bioinformatic methods. Ultimately, after bioinformatic analysis, key genes were obtained. These key genes might be common targets for treating both diseases, suggesting that the diseases share a common pathogenic mechanism. The mutual regulatory relationship among key genes, transcription factors, RNA-binding proteins and noncoding RNAs was predicted, and the up- and downregulation networks of key genes were constructed. These regulatory networks will help us further study the pathogenesis and early diagnosis of asthma and COVID-19.

## Materials and methods

### Data acquisition and processing

The National Center for Biotechnology Information (NCBI) Gene Expression Omnibus (GEO) database (http://www.ncbi.nlm.nih.gov/geo) is an open-access platform for data. It contains the most microarray chip and high-throughput sequencing gene expression profiling data to date. We downloaded second-generation high-throughput sequencing data for peripheral blood samples from 4 asthma patients and 3 healthy subjects (GSE195599) from GEO [21]. The GSE69683 dataset contains peripheral blood samples from 411 asthma patients and 87 healthy controls [22]. The GSE196822 dataset contains the gene expression profiles of 34 COVID-19 patients and 9 healthy controls. The GSE171110 dataset contains the whole-blood gene expression profiles of 44 COVID-19 patients and 10 healthy donors [23]. According to the annotation information on the platform, the probes are labeled with gene symbols, multiple probes corresponding to the same gene are randomly selected to remove duplicates, and then the gene expression matrix is obtained.

### Screening of differentially expressed genes

The “limma” package in R is a powerful method for analyzing differentially expressed genes (DEGs). We used it to screen DEGs between patients and controls in the GSE195599 and GSE196822 datasets. *p* < 0.05 and | log2-fold change (FC) |≥ 1 were set as the threshold values for DEG identification.

### GO and KEGG enrichment analyses

GO enrichment analysis divides gene functions into three categories: cellular component (CC), molecular function (MF) and biological process (BP). By using GO enrichment analysis, the regulatory relationships of target genes related to the CC, MF and BP categories could be obtained. KEGG enrichment analysis showed that genes were involved in multiple disease pathways, and gene functions were annotated. We used the “org.Hs.eg.db” package of R software for GO and KEGG enrichment analyses of differentially expressed genes to explore the shared pathogenesis between asthma and COVID-19.

### Protein‒protein interaction network analysis

Interactions between proteins can indicate the molecular mechanism of protein function. We constructed a protein–protein interaction (PPI) network using the STRING online protein interaction database (https://cn.string-db.org/). To further screen key genes, we used Cytoscape V 3.7.1 software to screen the 10 proteins with the strongest interactions in the PPI network. Key gene interaction regulatory protein networks were predicted by using the GENEMANIA online database (http://genemania.org/). The NetworkAnalyst online database (https://www.networkanalyst.ca/) was used to predict transcription factors that regulate key genes.

### LncRNA‒miRNA‒mRNA regulatory network construction

We used the “limma” package to screen differentially expressed lncRNAs between patients and controls in the GSE195599 and GSE196822 datasets. *p* < 0.05 and | log2-fold change (FC) |≥ 0.5 were set as the threshold values for differentially expressed lncRNA identification. The StarBase V3.0 online database (https://starbase.sysu.edu.cn/index.php) predicted miRNAs that might be regulated by lncRNAs. Funrich V2.0 software was used to analyze the signaling pathways that miRNAs might regulate. MiRNAs that regulate key genes were predicted by using the miRWalk database (http://mirwalk.umm.uni-heidelberg.de/). The lncRNA‒miRNA‒mRNA regulatory network was constructed with Cytoscape software.

### RBP–lncRNA regulatory network construction

The proteins that bind to RNA, known as RNA-binding proteins (RBPs), have powerful gene-regulating activity. We used the StarBase V3.0 database to predict RBPs that might regulate differentially expressed lncRNAs, and Cytoscape software was used to construct the RBP–lncRNA network.

### Key gene–oxidative stress-related protein interaction network construction

A list of oxidative stress-related proteins was downloaded from the GeneCards online database (https://www.genecards.org/). The top 20 genes with oxidative stress scores were selected to construct a regulatory network. The protein interaction network was built with the STRING online database, and the visual network was built with Cytoscape software.

### Validation of the roles of key genes in the diagnosis of COVID-19

An online receiver operating characteristic curve (ROC) plotting platform (https://www.cloudtutu.com/#/index) was used to construct ROC curves for the key genes in GSE196822 and GSE171110. The “Pheatmap” package was used to draw a heatmap of the key genes in GSE171110.

### Validation of the differential expression of key genes in asthma

The GSE69683 dataset contains sequenced RNA numbers of high flux in the peripheral venous blood of 87 healthy subjects and 411 patients with asthma. The “ggpubr” package in R was used to analyze differentially expressed genes.

### Cell culture

BEAS-2B cells were purchased from the Cell Bank of the Chinese Academy of Sciences. Cells were cultured in an incubator at 37 °C and 5% CO_2_ with RPMI 1640 medium (Gibco, Carlsbad, CA, USA) containing 10% fetal bovine serum (FBS; Gibco, Carlsbad, CA, USA).

### Asthma cell model construction

We used IL-4, IL-13 and IL-17A (Biosharp, AnHui, China) to construct an asthma cell model. Quantitative real-time PCR (qRT‒PCR) was used to detect common inflammatory factors to validate the model.

### Cell transfection

The experimental procedures for transfection assays followed the instructions for Lipofectamine (LEAGENE, Beijing, China). si-CXRC1 (Tsingke, Beijing, China) (sequence: 5′-CCCGCGTCACTTGGTCAAGTTTGT-3′) was transfected into cells. After 6 h, the medium was changed to RPMI 1640 medium supplemented with 10% FBS for continued culturing for 48 h.

### RNA extraction and qRT‒PCR

Total RNA was extracted using RNA-easy Isolation Reagent (Vazyme, Nanjing, China) and reverse transcribed into cDNA using a reverse transcription kit (Vazyme, Nanjing, China) according to the manufacturer's protocol. qRT‒PCR was performed following the instructions for the qRT‒PCR reagents (Vazyme, Nanjing, China). The primers were as follows: CXRC1, 5′-CCAGGCTTACCATCCAAACAAT-3′ and 5′-GCAGGGTGAATCCATAGCAGAAC-3′; TSLP, 5′-AATCGGCCACATTGCCTTAC-3′ and 5′-CATGGCGAACATTTCTTTGG-3′; IL-25, 5′-TGAGGGAGCGACCCAGATTA-3′ and 5′-GCCAAGAATGCAACCACCTG-3′; and β-actin, 5′-CACAGAGCCTCGCCTTTGC-3′ and 5′-ACCCATGCCCACCATCACG-3′.

### Cell counting kit-8 (CCK-8) assay

Cells were seeded at a density of 3.0 × 10^3^ cells per well in a 96-well plate, and each group included three replicate wells. Ten microliters of CCK-8 (Sigma‒Aldrich, St. Louis, MO, USA) was added to each well at 24 h, 48 h and 72 h. After a 4-h incubation, the absorbance at 450 nm of each well was measured on a microplate reader.

### Statistical analysis

R studio software V3.1.3 and Cytoscape V3.7.1 software were used to analyze public gene expression data. GraphPad V8.0.2 software was used for statistical analysis of clinical data. All data were compared between two groups as the mean ± standard deviation for continuous-variable data, and a t test was used for comparison. Differences were considered statistically significant at *p < 0.05, **p < 0.01, and ***p < 0.001.

## Results

### Identification of common differentially expressed mRNAs between asthma and COVID-19

**Fig. 1:**
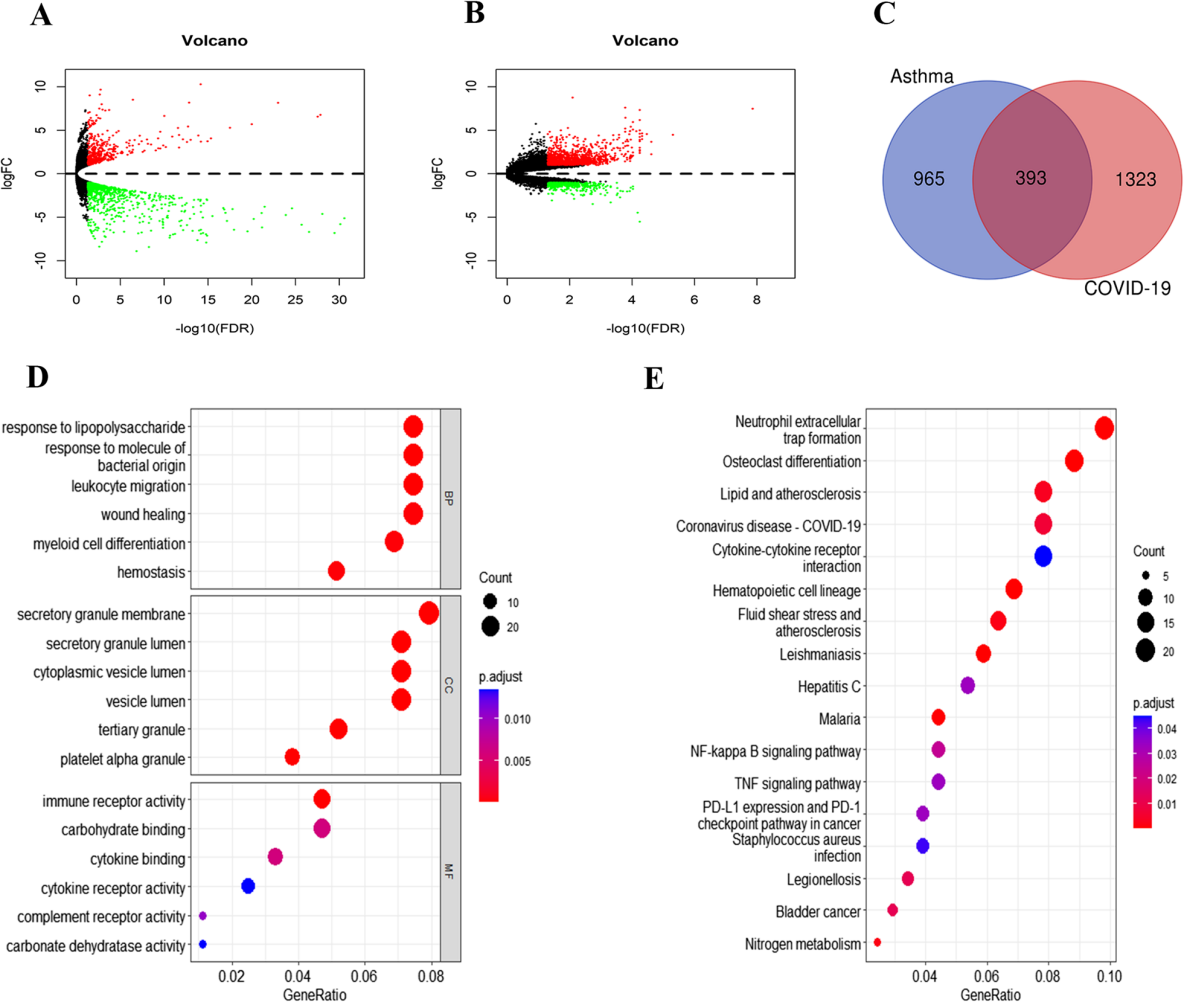
**A** Volcano plot of differentially expressed mRNAs in the GSE195599 dataset. **B** Volcano plot of differentially expressed mRNAs in the GSE196822 dataset. **C** Common differentially expressed mRNAs between GSE195599 and GSE196822. **D** GO enrichment analysis of common differentially expressed mRNAs. **E** KEGG enrichment analysis of common differentially expressed mRNAs

To study the relationship and interaction between asthma and COVID-19, we downloaded and analyzed the GSE195599 and GSE196822 datasets from the GEO database. There were 1358 differentially expressed mRNAs in the GSE195599 asthma dataset, of which 478 were upregulated and 880 were downregulated (Fig. 1A). Among the 1716 differentially expressed mRNAs in the GSE196822 COVID-19 dataset, 1269 were upregulated, and 447 were downregulated (Fig. 1B). We identified 393 common differentially expressed mRNAs between the asthma and COVID-19 datasets (Fig. 1C).

### GO and KEGG enrichment analyses

We performed GO enrichment analysis and KEGG enrichment analysis of the 393 common differentially expressed mRNAs. GO enrichment analysis showed that the differentially expressed mRNAs were mainly concentrated in response to lipopolysaccharide, secretory granule and immune receptor activity (Fig. 1D). KEGG enrichment analysis showed that the differentially expressed mRNAs were mainly enriched in neutrophil extracellular trap formation, osteoclast differentiation and lipid and atherosclerosis (Fig. 1E).

### Protein‒protein interaction network analysis

**Fig. 2:**
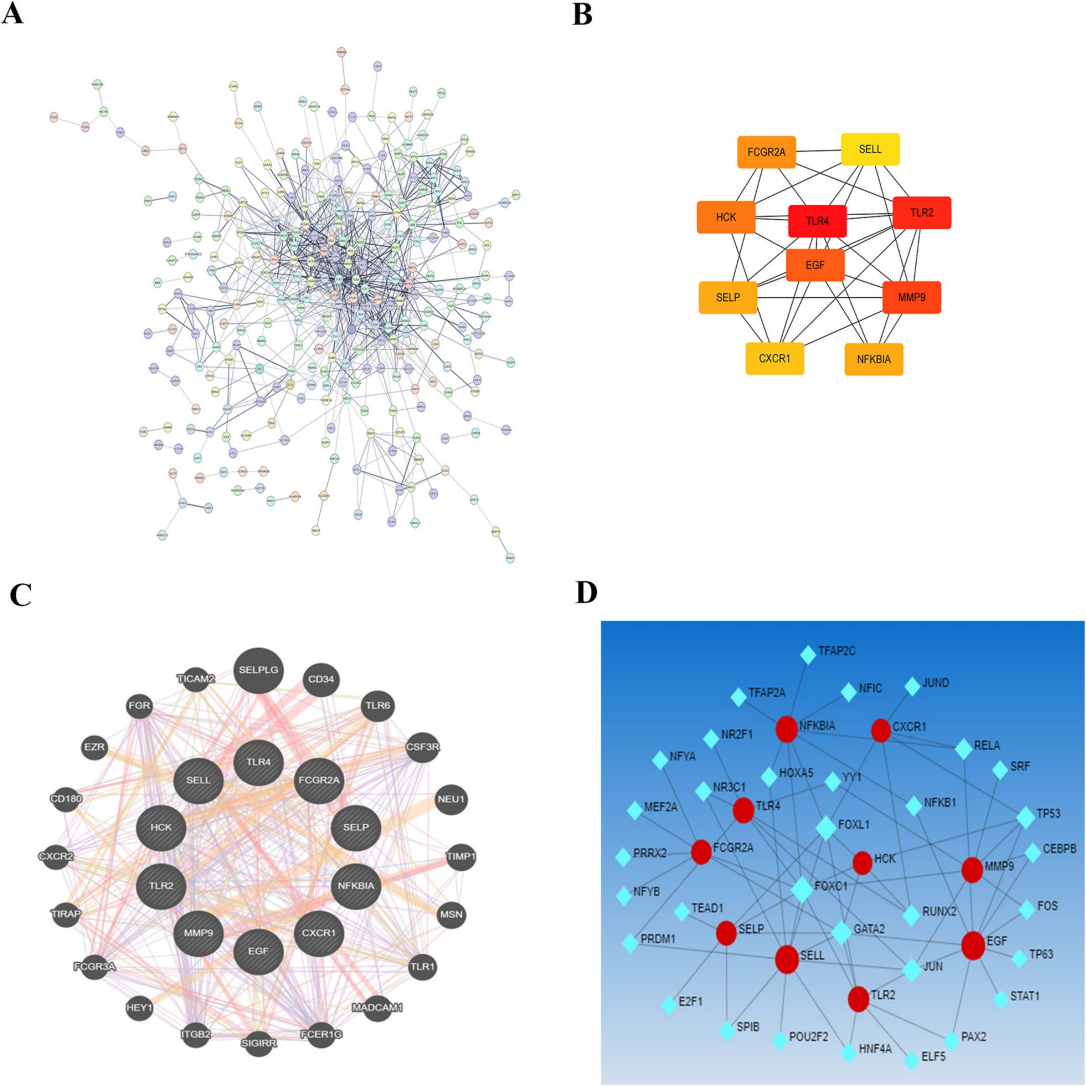
**A** Protein interaction network of common differentially expressed mRNAs. **B** Interaction network of key genes. **C** Interaction network of key genes and proteins regulated by the key genes. **D** Interaction network of key genes and transcription factors that regulate the key genes

We constructed a protein interaction network with the STRING database (Fig. 2A and Additional file 1: Fig. S1). Cytoscape software was used to analyze the protein–protein interaction network, and key genes (TLR4, TLR2, MMP9, EGF, HCK, FCGR2A, SELP, NFKBIA, CXCR1, and SELL) were screened out (Fig. 2B). These key genes might be biomarkers for both asthma and COVID-19. Interacting proteins regulated by the key genes were predicted by the GENEMANIA database (Fig. 2C). The NetworkAnalyst database was used to predict transcription factors that regulate the key genes (Fig. 2D).

### Construction of an lncRNA‒miRNA–mRNA regulatory network

**Fig. 3:**
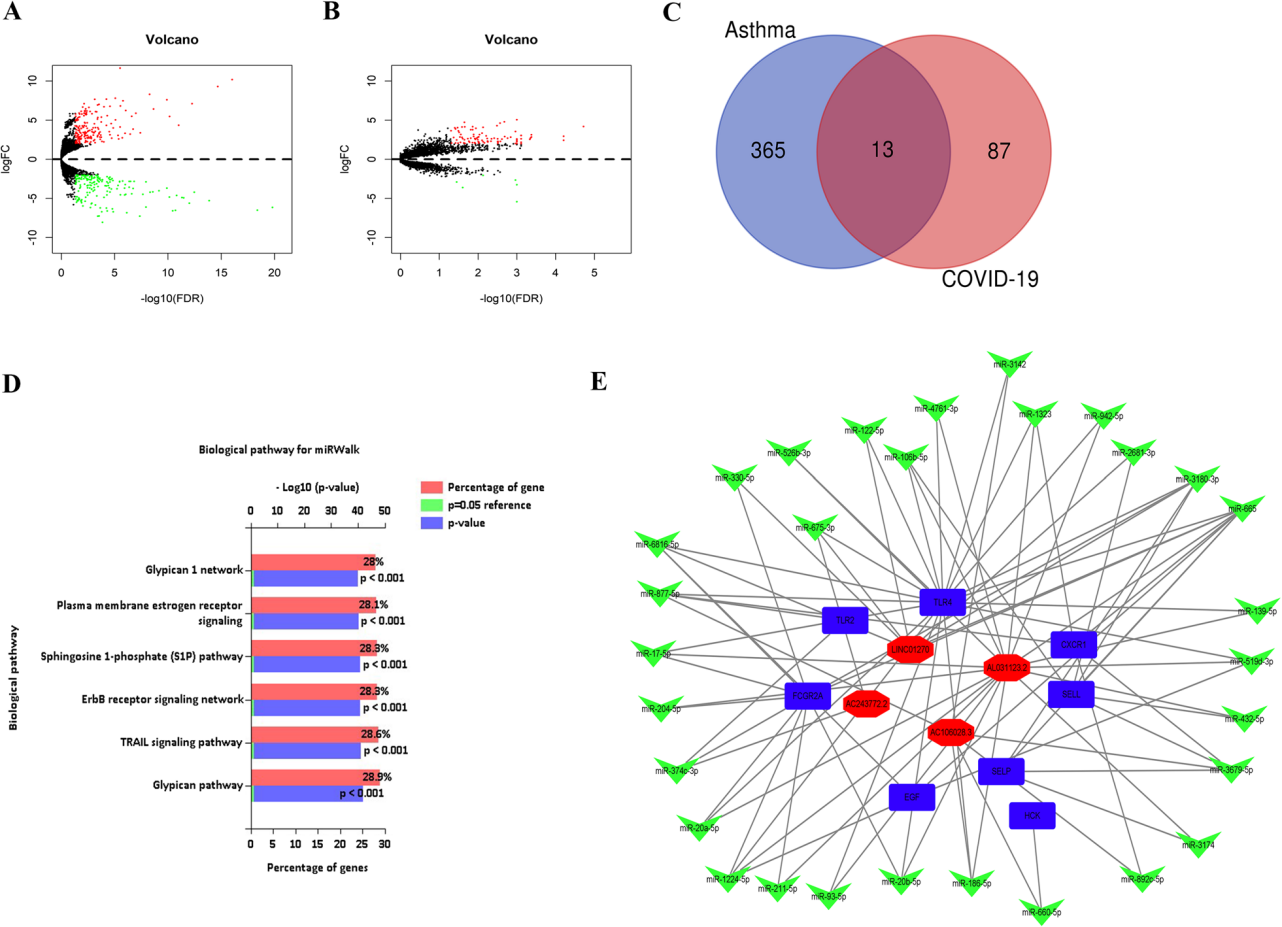
**A** Volcano plot of differentially expressed lncRNAs in the GSE195599 dataset. **B** Volcano plot of differentially expressed lncRNAs in the GSE196822 dataset, **C** Common differentially expressed lncRNAs between the GSE195599 and GSE196822 datasets. **D** KEGG enrichment analysis of miRNAs regulated by common differentially expressed lncRNAs. **E** LncRNA–miRNA‒mRNA regulatory network

To construct an lncRNA‒miRNA–mRNA regulatory network, we downloaded and analyzed the GSE195599 and GSE196822 datasets from the GEO database. There were 507 differentially expressed lncRNAs in asthma, of which 252 were upregulated and 255 were downregulated (Fig. 3A). Among the 304 differentially expressed lncRNAs in the COVID-19 dataset, 248 were upregulated, and 56 were downregulated (Fig. 3B). We identified 13 common differentially expressed lncRNAs between the asthma and COVID-19 datasets (Fig. 3C). We predicted differentially expressed lncRNA-regulated miRNAs with the StarBase V3.0 database and used Funrich software to analyze the signaling pathways that the miRNAs might regulate (Fig. 3D). The miRWalk database was used to predict the miRNAs that regulate the key genes, and Cytoscape software was used to construct the lncRNA–miRNA–mRNA regulatory network (Fig. 3E).

### An RBP–lncRNA regulatory network was constructed

**Fig. 4:**
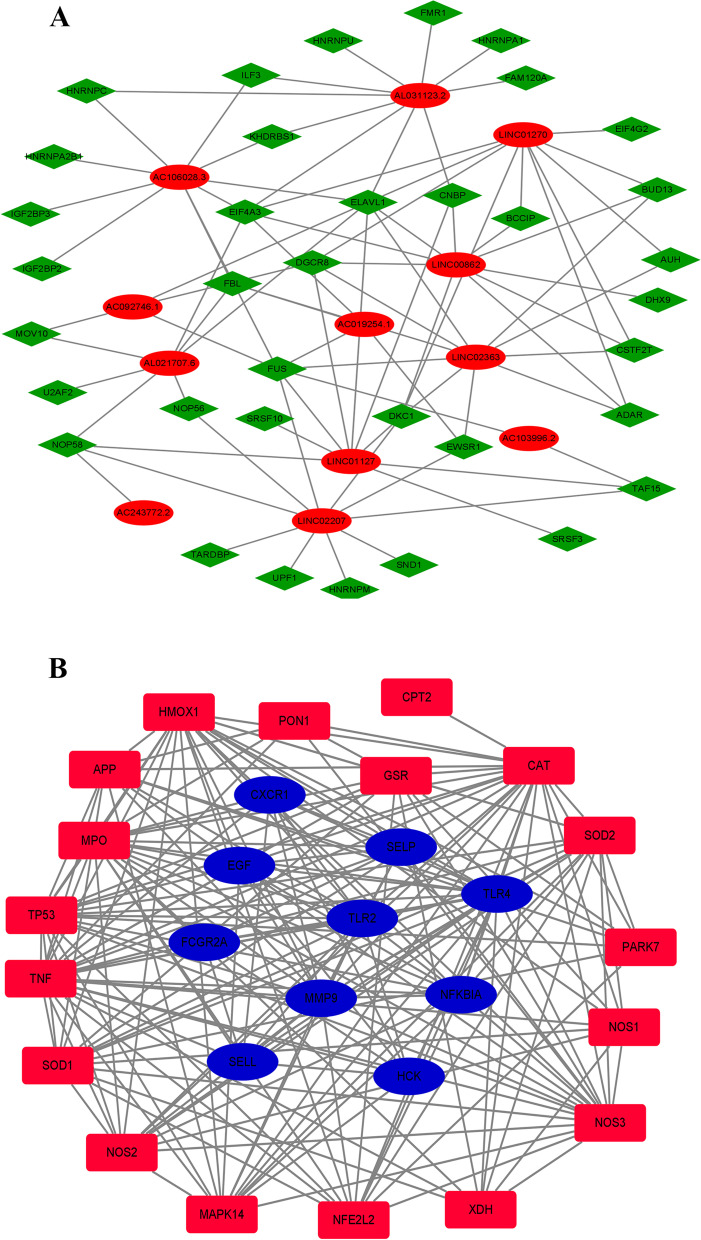
**A** RBP–lncRNA regulatory network. **B** Interaction network of key genes and oxidative stress-related proteins

We used the StarBase V3.0 database to predict RBPs that might regulate the differentially expressed lncRNAs and Cytoscape software to construct the RBP–lncRNA network (Fig. 4A).

### An interaction network connecting the key genes and oxidative stress-related proteins was constructed

A list of oxidative stress-related proteins was downloaded from the GeneCards online database. The top 20 genes with oxidative stress scores were selected to construct a regulatory network (Fig. 4B).

### Validation of the roles of key genes in the diagnosis of COVID-19

**Fig. 5:**
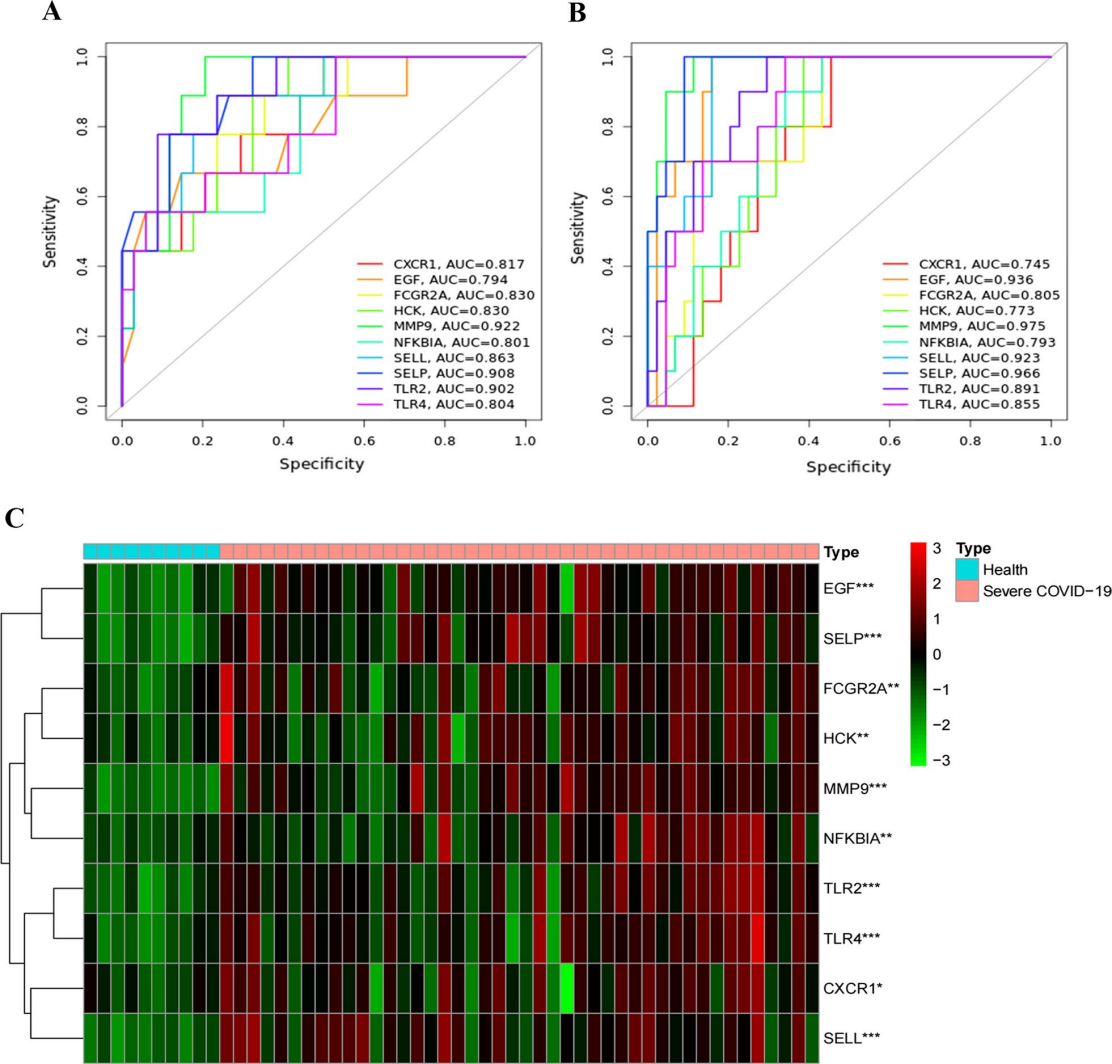
**A** ROC curves of key genes in GSE196822. **B** ROC curves of key genes in the validation set GSE171110. **C** Heatmap of key genes in the validation set GSE171110

ROC curves for the key genes in GSE196822 and GSE171110 were plotted using an online website (Fig. 5A, B). The “Pheatmap” package was used to draw a heatmap of the key genes in GSE171110 (Fig. 5C).

### Validation of the differential expression of key genes in asthma

**Fig. 6:**
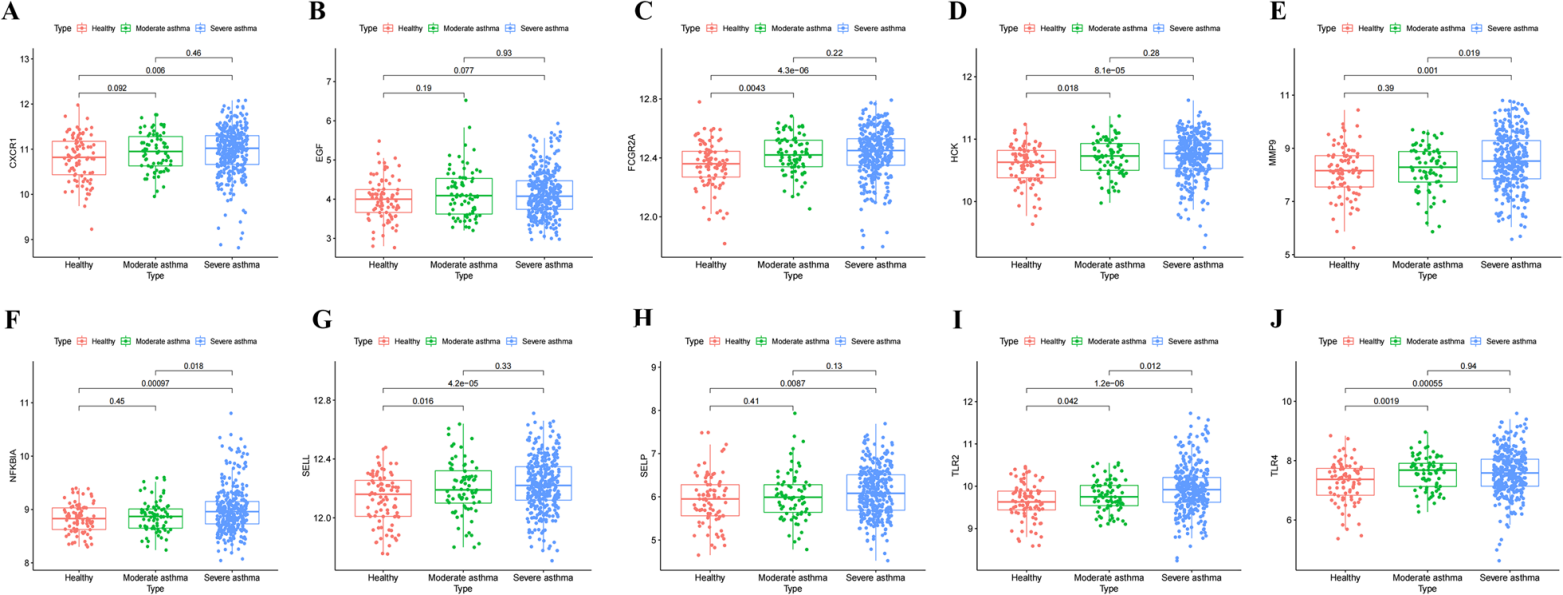
The expression of key genes in the GSE69683 dataset

We analyzed the GSE69683 dataset to verify the differential expression of key genes (Fig. 6).

### Preliminary laboratory validation of the key genes in a cell model of asthma

**Fig. 7:**
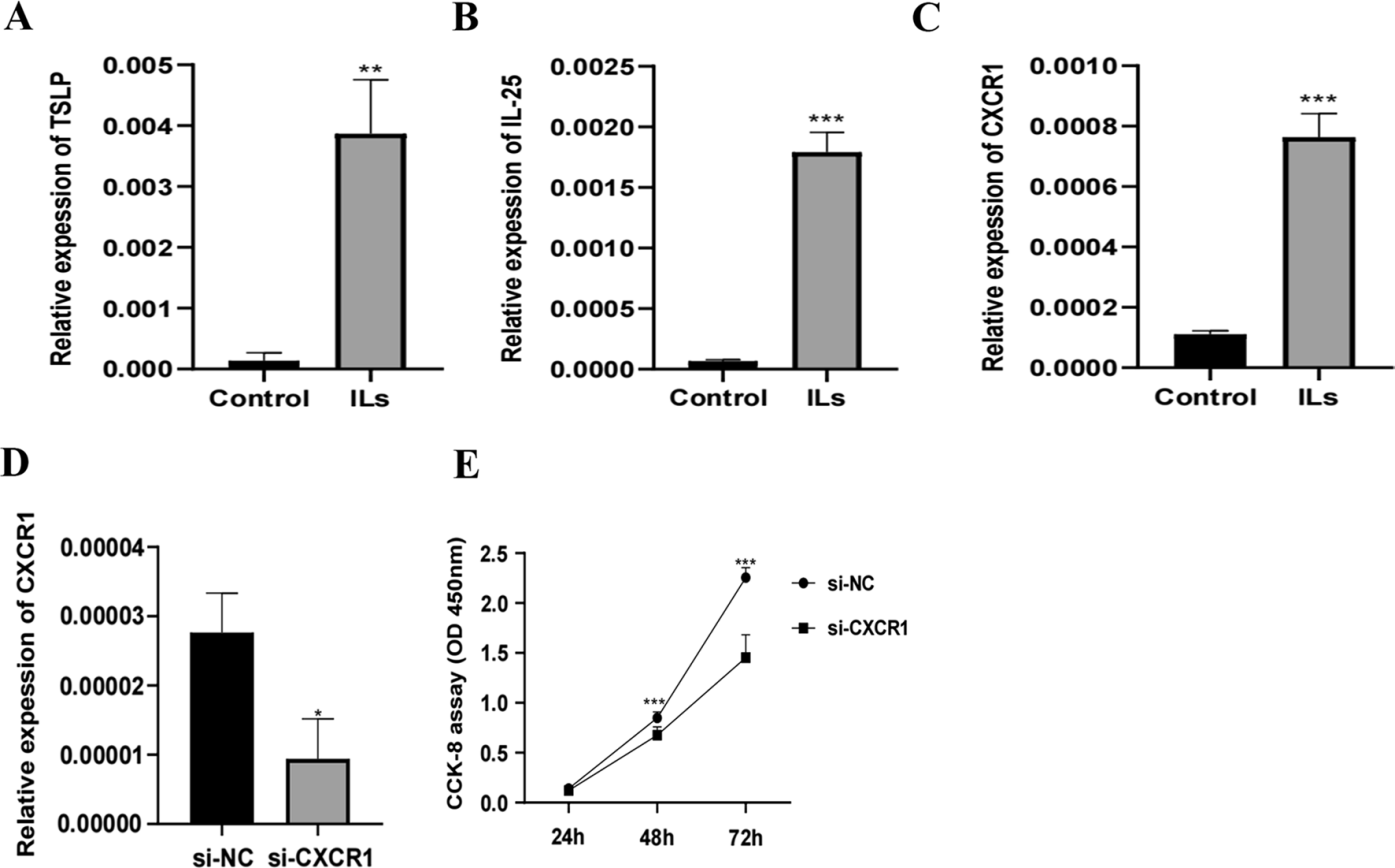
A, B The expression of the inflammatory markers TSLP and IL-25 (**p < 0.01, ***p < 0.001). **C** The expression of CXRC1 (***p < 0.001). **D** Interference with CXRC1 expression in BEAS-2B cells (*p < 0.05). **E** CCK-8 assay detecting changes in BEAS-2B cell proliferation (***p < 0.001)

We stimulated BEAS-2B cells with IL-4, IL-13 and IL-17A to simulate the asthma microenvironment. The expression of the inflammatory markers TSLP and IL-25 was significantly upregulated in the experimental group (Fig. 7A, B). We selected CXCR1 for preliminary laboratory validation (Fig. 7C). The expression of CXCR1 was silenced with siRNA (Fig. 7D). A CCK-8 assay showed that the viability of BEAS-2B cells was reduced considerably after transfection of si-CXCR1 (Fig. 7E).

## Discussion

Molecular biology mainly elucidates the occurrence, development and influencing factors of diseases at a molecular level, and it can often determine the biomarkers of diseases in clinical application. The development of computers has made it possible to analyze high-throughput data generated with this approach [24]. The application of bioinformatics has enriched the diversity of disease research and analysis methods [25].

Asthma is a lung disease characterized by reversible airway obstruction, airway inflammation and increased airway responsiveness to multiple stimuli. Asthma is associated with polygenic inheritance and affected by both genetic and environmental factors [26]. COVID-19 is an infectious disease caused by SARS-CoV-2 that has reached pandemic proportions. Overactivated natural immunity and “cytokine storm” are considered to be the underlying pathological mechanisms for the rapid progression of COVID-19 [27]. Currently, the interaction between the two is not clear, but several studies have shown that the two are closely related. Children and adults with severe or poorly controlled asthma are at an increased risk of hospitalization following novel coronavirus infection, according to a new study using the OFFICE for National Statistics. Researchers found that among adults, people who used low doses of inhaled steroids for asthma control were less likely to be hospitalized or die from COVID-19 than people without asthma [28]. In theory, asthmatic patients should have an increased sensitivity of SARS-CoV-2 infection and increased infection severity because of the lack of an antiviral immune response and the tendency for deterioration caused by common respiratory viruses [29].

We obtained key genes related to pathogenesis shared between asthma and COVID-19 through bioinformatic methods. The results showed that TLR4, TLR2, MMP9, EGF, HCK, FCGR2A, SELP, NFKBIA, CXCR1, and SELL were closely related to asthma and COVID-19. TLR4 is a type I transmembrane protein that recognizes lipopolysaccharide and initiates intracellular signal transduction via the NF-κB or JNK/SAPK signaling pathways [30]. Researchers found that TLR2 and MYD88 expression were both associated with the severity of COVID-19. Mechanistically, TLR2 and MYD88 were required for β-coronavirus-induced inflammation, and TLR2-dependent signaling pathways induced proinflammatory cytokine production during coronavirus infection independent of viral entry. TLR2 sensed the SARS-CoV-2 envelope protein as its ligand. In addition, blocking TLR2 signaling in vivo provided protection against the pathogenesis of SARS-CoV-2 infection. This study provided important insights into the molecular mechanisms underlying β-CoV sensing and inflammatory cytokine production, opening new avenues for the development of therapeutic strategies for COVID-19 [31]. MMP9 is the largest enzyme in the matrix metalloproteinase family. It is secreted as a zymogen and activated to form type IV collagenase. MMP9 can degrade and destroy types IV and V collagen and gelatin in the extracellular matrix near the tumor surface; subsequently, tumor cells can infiltrate the surrounding tissues through the disrupted basement membrane, eventually leading to tumor invasion and metastasis [32]. EGF is a growth factor that stimulates cell growth, proliferation and differentiation by binding to its receptor, EGFR [33]. A previous study found that the expression level of EGFR in bronchial epithelial cells was increased in asthma and correlated with disease severity and that the activation of EGFR signaling in airway epithelial cells contributed to mucus metaplasia in a chronic asthma model [34]. HCK is closely related to hematopoietic function and may play important roles in neutrophil migration and degranulation [35]. FCGR2A encodes a member of a family of immunoglobulin Fc receptor genes expressed on the surface of many immune response cells [36]. The protein encoded by this gene is a cell-surface receptor found on phagocytic cells, such as macrophages and neutrophils, and is involved in the processes of phagocytosis and immune complex clearance [37]. CXCR1 is able to bind to the inflammatory cytokine IL-8 and trigger a cascade of events through intracellular G proteins that activate multiple signaling pathways [38]. Through bioinformatic analysis, researchers conducted an in-depth analysis of alveolar lavage fluid datasets for COVID-19 and asthma. Ultimately, EEF1A1, EGR1, UBA52, DDX5 and IRF8 might be the key shared pathogenesis-related genes in COVID-19 and asthma. Moreover, LY294002, wortmannin, PD98059 and heparin were found to be potential agents for treating COVID-19 in the context of asthma [39]. The research method of this study was similar to ours, but the results were different. It is speculated that different datasets might come from different tissue samples. This suggests that we still need large-sample and multicenter related studies.

In conclusion, COVID-19 is a newly discovered disease, and there is not much research on its risk factors or disease process [40]. Currently, a number of vaccines are available to prevent COVID-19. However, the vaccines have not been shown to be effective in some cases, particularly against different variants of SARS-CoV-2 [41]. Our study performed transcriptome analysis to detect pathways and molecular biomarkers common between asthma and COVID-19, helping to improve the understanding of the association between asthma and COVID-19. Ten hub proteins were found in these diseases. Therefore, the genes we identified could be new therapeutic targets for COVID-19 vaccine development.

## Supplementary Information

**Additional file 1: Fig. S1**. Protein interaction network of common differentially expressed mRNAs.

## Data Availability

The datasets used and/or analyzed in the current study are available from the corresponding author on reasonable request.

## Abbreviations

SARS-CoV-2: Severe acute respiratory syndrome coronavirus 2
COVID-19: Causes coronavirus disease 19
GEO: Gene Expression Omnibus
GO: Gene Ontology
KEGG: Kyoto Encyclopedia of Genes and Genomes
lncRNAs: Long noncoding RNAs
miRNA: MicroRNA
ceRNA: Competing endogenous RNA
NCBI: National Center for Biotechnology Information
DEGs: Differentially expressed genes
CC: Cellular component
MF: Molecular function
BP: Biological process
PPI: Protein‒protein interaction
RBPs: RNA-binding proteins
ROC: Receiver operating characteristic
FBS: Fetal bovine serum
qRT‒PCR: Quantitative real-time PCR
CCK-8: Cell Counting Kit-8
