# Supplementary figures and images for: Bioinformatic analysis and preliminary validation of potential therapeutic targets for COVID-19 infection in asthma patients
Source: Cell Commun Signal. 2022 Dec 27;20:201. doi: 10.1186/s12964-022-01010-2 (PMC9793391; doi:10.1186/s12964-022-01010-2)

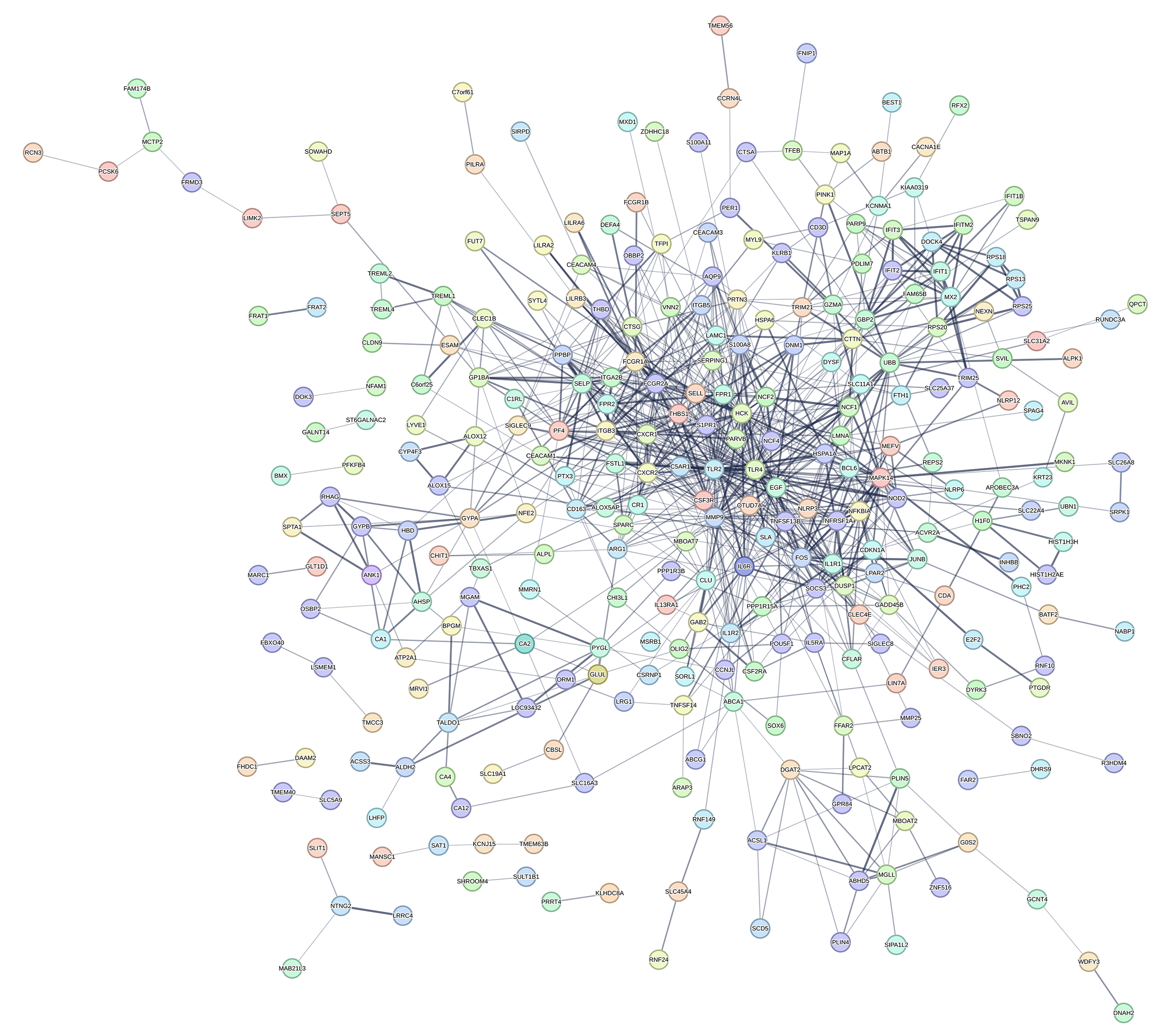

Supplement: Supplementary file 2 — Additional file 1: Fig. S1. Protein interaction network of common differentially expressed mRNAs. [file 12964_2022_1010_MOESM2_ESM.tif]
